# Wiring and Volume Transmission: An Overview of the Dual Modality for Serotonin Neurotransmission

**DOI:** 10.1021/acschemneuro.3c00648

**Published:** 2023-11-15

**Authors:** Giulia Gianni, Massimo Pasqualetti

**Affiliations:** † Unit of Cell and Developmental Biology, Department of Biology, University of Pisa, 56127 Pisa, Italy; ‡ Center for Neuroscience and Cognitive Systems @UniTn, Istituto Italiano di Tecnologia, 38068 Rovereto, Italy; § Centro per l’Integrazione della Strumentazione Scientifica dell’Università di Pisa (CISUP), 56126 Pisa, Italy

**Keywords:** Serotonin, serotonergic fibers, volume transmission, wiring transmission, synapse, nonjunctional varicosity

## Abstract

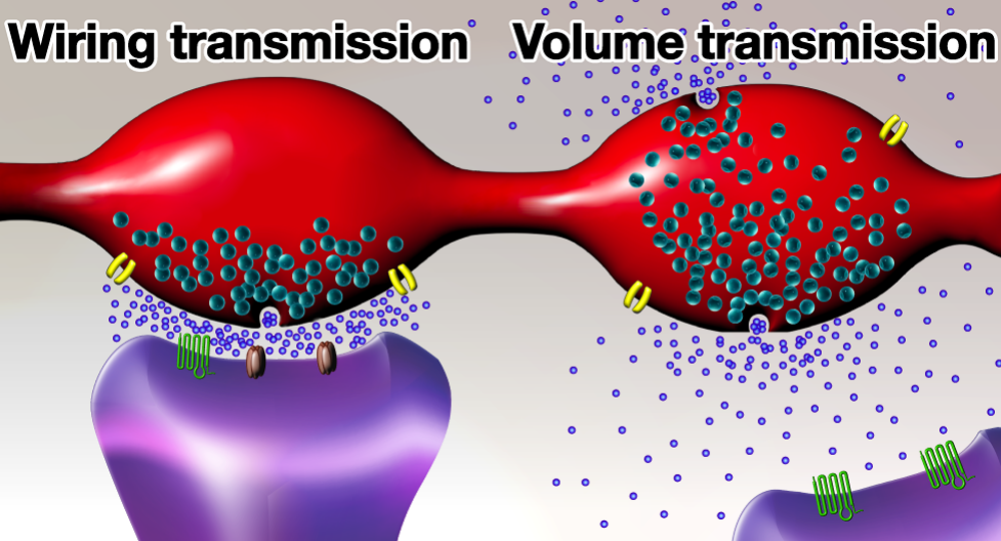

Serotonin is a neurotransmitter involved in the modulation of a multitude of physiological and behavioral processes. In spite of the relatively reduced number of serotonin-producing neurons present in the mammalian CNS, a complex long-range projection system provides profuse innervation to the whole brain. Heterogeneity of serotonin receptors, grouped in seven families, and their spatiotemporal expression pattern account for its widespread impact. Although neuronal communication occurs primarily at tiny gaps called synapses, wiring transmission, another mechanism based on extrasynaptic diffusion of neuroactive molecules and referred to as volume transmission, has been described. While wiring transmission is a rapid and specific one-to-one modality of communication, volume transmission is a broader and slower mode in which a single element can simultaneously act on several different targets in a one-to-many mode. Some experimental evidence regarding ultrastructural features, extrasynaptic localization of receptors and transporters, and serotonin–glia interactions collected over the past four decades supports the existence of a serotonergic system of a dual modality of neurotransmission, in which wiring and volume transmission coexist. To date, in spite of the radical difference in the two modalities, limited information is available on the way they are coordinated to mediate the specific activities in which serotonin participates. Understanding how wiring and volume transmission modalities contribute to serotonergic neurotransmission is of utmost relevance for the comprehension of serotonin functions in both physiological and pathological conditions.

## Introduction

The mammalian central nervous system (CNS) has an extremely complex organization. The human brain is estimated to contain around 86.1 billion neurons and a similar number of glial cells;^1^ only in the neocortex the number of synapses is evaluated to be around 164 trillion,^2^ and in the whole adult CNS there might be over 10^15^ synaptic contacts.^3^ In light of this, synaptic communication is reasonably recognized as the principal modality through which information is processed and elaborated. The complexity of this system increases further, taking into account the high variability of the neurons that compose the CNS, each one characterized by unique combinations of morphological, neurochemical, electrophysiological, and hodological properties.

In this framework, the serotonergic system stands out due to some peculiar characteristics. Serotonin (5-hydroxytryptamine, 5-HT) producing neurons constitute a relatively small fraction of the total neurons in the CNS. In fact, it is estimated the presence of approximately 300 000 serotonergic cells in the human brain and only around 26 000 over a total of 70 million neurons in the most widely used mammalian animal model, the mouse.^4−7^ 5-HT neurons are among the earliest generated during development, differentiating at mid-gestation along the midline of the rhombencephalon and subsequently migrating to defined areas of the brainstem where they integrate within the raphe nuclei, clustered in the B1–B9 groups.^8,9^ In spite of the limited number of serotonin-producing neurons, brain serotonin has been shown to be involved in the modulation of a broad range of different physiological and behavioral processes, including regulation of circadian rhythms, mood, feeding behavior, and social interaction.^10−12^ Moreover, prior to its activity as a neurotransmitter in the mature brain, serotonin has been shown to play a crucial role in development and plasticity as it influences processes including cell proliferation and migration, neuronal differentiation, and circuit formation.^9,13−17^ Accordingly, alteration of the normal serotonergic neurotransmission is associated with the emergence of psychiatric disorders that are thought to originate during development.^18−21^

A major factor in determining the capacity of serotonin to modulate such a vast multitude of diverse functions lies in the extensive innervation that the serotonergic neurons provide to the brain so that virtually each area receives 5-HT innervation. In fact, their projections spread throughout the CNS, from the anteriormost parts of the telencephalon to the spinal cord, forming an ascending system, responsible for the innervation of the forebrain and mostly originating from the rostral group (B5–B9) and a descending system, arising from the caudal group (B1–B4).^22^

Another key point concerns the multiplicity of 5-HT receptors, which are both ionotropic and metabotropic. In mammals, 14 serotonin receptors are known and they are organized in seven families (5-HT_1–7_), distinguished on the basis of pharmacological profiles, signal transduction pathways, and structural characteristics.^23,24^ In addition, the high spatiotemporal variability of their expression plays a crucial role in determining the specificity of the 5-HT effects.

The heterogeneity of 5-HT neurons represents an additional aspect to consider. In spite of being originally defined exclusively by their shared serotonergic phenotype, there is growing evidence for the existence of distinct populations of 5-HT neurons which differ in their molecular identities and functional properties, concerning traits such as gene expression, electrophysiology, connectivity, and neurochemistry.^25,26^

**Figure 1:**
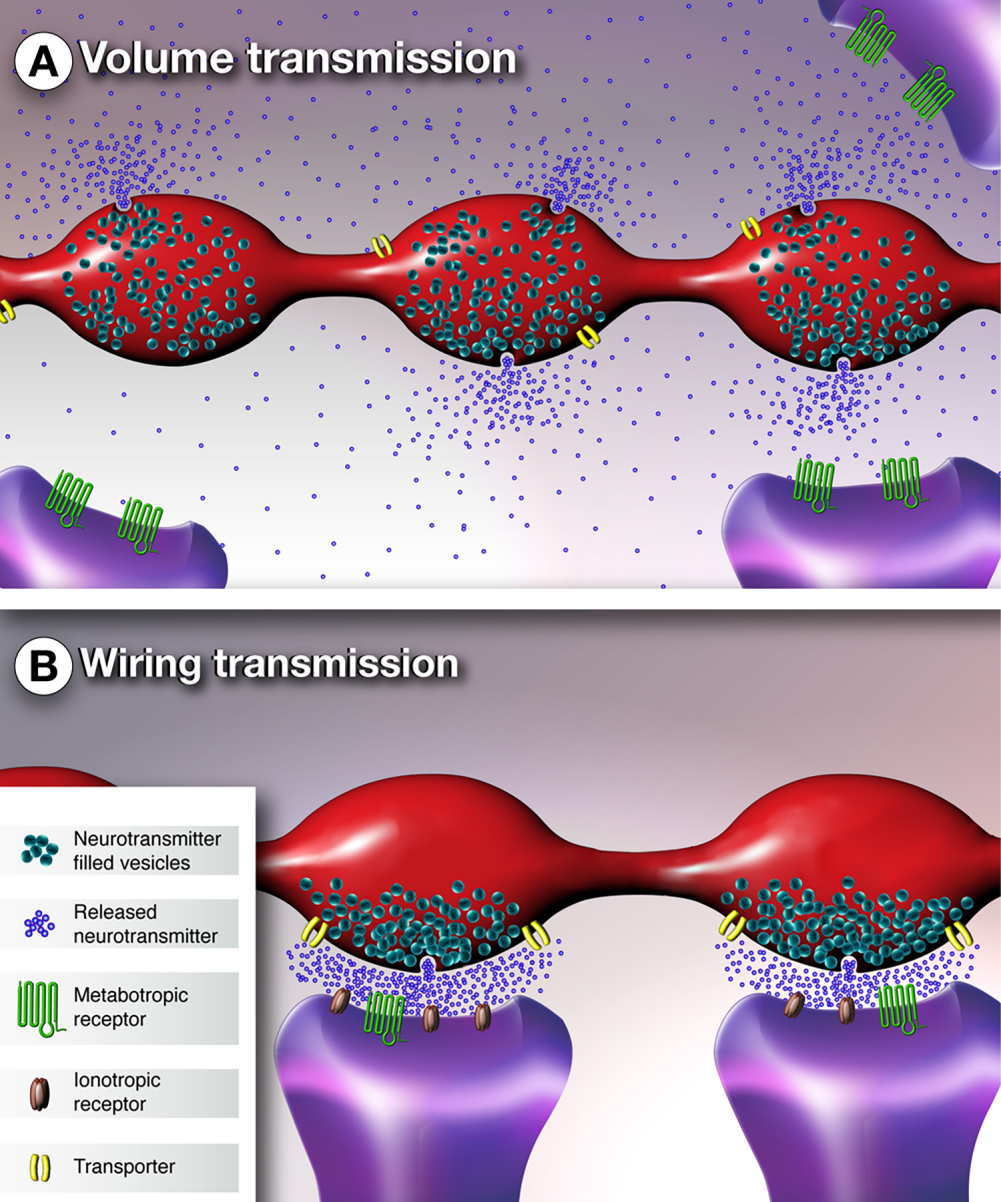
Schematic representation of volume and wiring transmission. (A) Simplified axonal varicosities displaying volume transmission in which the neurotransmitter is released in the extracellular space, where it diffuses and reaches metabotropic receptors located on nearby cells. (B) Simplified axonal varicosities showing wiring transmission. Each varicosity establishes a synaptic contact with specific postsynaptic element, and the neurotransmitter is released in a confined space, where it interacts with both ionotropic and metabotropic receptors.

In contrast to the availability of extensive amounts of data concerning the characteristics of serotonergic transmission described so far, there is another aspect that appears to be much less explored, despite its potential relevance in determining the widespread effects of serotonin. In fact, besides the canonical synaptic exchange of information, another important modality of intercellular communication for monoaminergic systems is present. This modality, based on the diffusion of chemical signals in the extracellular space, is referred to as volume transmission (Figure 1A).^27,28^

Though it is commonly accepted that the 5-HT system relies both on the more conventional synaptic wiring transmission and on volume transmission, in the literature there is a limited body of evidence supporting the actual presence of the latter mechanism and, from a functional point of view, little is known about the role of each of the two. Starting from general considerations on the two modalities of intercellular communication in the CNS, in this review we will focus on the existing experimental evidence supporting the presence of nonsynaptic volume transmission in the serotonergic system.

Deciphering the mechanisms controlled by either wiring or volume transmission and the cascade of events that each of the two modalities promotes is critical to fully understand the action of serotonin in regulating the myriad of functions in which it is involved.

## Intercellular Communication Modalities in the CNS

In the central nervous system, interneuronal communication is mainly associated with the activity of synapses. Historically, the study of synapses has had significant relevance in advancing our comprehension of how neurons communicate. In this sense, the introduction of the “neuron doctrine” marked a fundamental step toward the understanding of synaptic communication and, in general, of the functioning of the nervous system.^29^ This theory, proposed by Santiago Ramon y Cajal, asserted the contiguity in between neurons, in contrast to the relationship of physical continuity postulated by the “reticular theory”, which has been popular throughout the 19th century and had among its supporters Camillo Golgi. The notion of neurons as anatomically and functionally independent units implied the existence of modalities of intercellular communication relying on extracellular signals. In the same years, the term synapse has been used for the first time by Charles Sherrington,^30^ who derived it from the Greek verb “synaptein” (συν “with”, α̋πτειν “to touch”) in order to highlight the existence of a point of contact between neurons as physically distinct elements. However, due to limitations of light microscopy in resolving the separation between synaptic elements, the existence of synapses remained speculative until the 1950s, when advances in electron microscopy enabled one to describe finely their structure, confirming the presence of the synaptic cleft between presynaptic and postsynaptic elements.^31,32^

Although synapses are still considered to play a predominant role, the importance of other modalities of intercellular communication among neurons has started to be appreciated, revealing the existence of an even more complex way of action for neurotransmitters that actually might represent the ancestral modality of communication used by neurons in the early days of nervous system evolution.^33^ The concept of volume transmission refers to a model introduced in the 1980s,^34^ according to which neurochemical communication in the brain can be distinguished in two main categories, termed wiring transmission and volume transmission, acting alongside to define the activity and shape the output of neural circuits (Figure 1).

Wiring transmission (WT) can be defined as a point-to-point communication, relying on the presence of well-defined structures through which the signals are transmitted, as virtual wires connecting two specific elements (Figure 1B).^28^ Chemical synapses represent a prototype for this kind of communication. As the axon potential reaches the presynaptic terminal, through a Ca^2+^-dependent exocytotic mechanism the neurotransmitter is released in the synaptic cleft and it diffuses across few nanometers (≈20 nm) to bind and activate receptors on the postsynaptic membrane.^35^ Besides chemical synapses, also gap junctions, or electrical synapses, constitute an integral part of WT, which can be found in the mammalian CNS.^36^ The functional properties of WT are closely tied to the structural characteristics of synapses. It is a fast and highly specific communication modality in which the source of the signal and its target are in a 1:1 ratio. The presence of physically defined structures ensures relative stability in the connection between the source of the signal and the target. Thanks to those features, WT appears to be particularly well suited for prompting activation or inhibition of effector systems as well as for guaranteeing an oriented flux of information through those “hardwired” networks. Volume transmission (VT), in contrast, defines the release and diffusion of neuroactive molecules within the volume of extracellular fluids in brain parenchyma, without defined physical restraints (Figure 1A).^28^ The capacity of a molecule of diffusing in the extracellular space depends on its energy gradients, and it is also strongly influenced by the characteristics of the extracellular space (ECS) itself. In particular, three parameters are important in defining the migration properties of a VT signal.^37^ The first of them is represented by the volume fraction being the size of the ECS with respect to the volume of the whole tissue, on average estimated around 20%. The second is the tortuosity as the increase in the extension of the path that a signal has to move through, dependent on the complexity of the tridimensional structure of the ECS. Finally, the clearance that is the rate of removal of the signaling molecule from the ECS itself.

The characteristics of VT make it profoundly different from classical synaptic communication. One significant distinction can be identified in its broad reach, allowing a signal to simultaneously act on many different elements in a relatively wide area, as long as they are competent to respond. In this way there is a shift from the one-to-one relationship between the source of the message and the target that characterizes synaptic communication to a one-to-many relationship in volume transmission. Furthermore, neurons are not the only cell types involved in this kind of interaction, but also glial cells present in the CNS can be both a source and a target for those extrasynaptic signals.^38,39^ On the other hand, the wider spatial distance over which the signal diffuses in VT results in a slower transmission compared to WT. Those properties make the VT appropriate for long-lasting modulatory functions that do not necessarily require a quick activation or inhibition.

As volume transmission is a neurochemical modality of intercellular communication, some requirements must be met for it to occur. First, it should be possible for the neurotransmitter to reach the extracellular space, which can occur through different mechanisms such as direct extrasynaptic exocytosis, synaptic spillover, or reverse activity of transporters.^40−42^ As a second point, a signal-decoding system should be present outside the synapses, meaning that extrasynaptic receptors should be located on target cells.^43^ Moreover, since receptors have specific affinity for their ligands, it is necessary that they are reached by a sufficiently high concentration of the signaling molecule in order to be effectively activated and generate a response. Finally, the presence of a mechanism to remove the neurotransmitter from the extracellular space is required in order to interrupt the signaling, such as functional extrasynaptic transporters. Evidence regarding the fulfillment of those requirements has been collected within the CNS in relation to serotonergic neurotransmission, indirectly suggesting the presence of VT mechanisms.^43−45^

## Ultrastructural Evidence Supporting Volume Transmission in the Serotonergic System

The introduction and the development of the concept of volume transmission are strictly linked to considerations concerning the characteristics of the central monoaminergic systems.^45^ In this regard, particularly significant has been the analysis of their ultrastructural features.

The central serotonergic system has been first identified and described in the mid-1960s.^8^ This initial characterization made use of the Falck–Hillarp method, a histochemical fluorescence technique based on the capacity of serotonin and catecholamines to react with formaldehyde forming products with defined fluorescent properties.^46,47^ Subsequently, the development of more sensitive and specific techniques made it possible to investigate the characteristics of 5-HT producing neurons and their projections within the CNS with a higher level of detail. Early studies employed autoradiographic techniques following tritiated 5-HT administration in vivo. Thanks to advances in immunohistochemistry, this method has then been largely replaced by immunostaining using antibodies against 5-HT itself,^48^ its biosynthetic enzyme tryptophan hydroxylase (TPH),^49^ or the serotonin transporter SERT.^50^

Over the years, those methods have been used to explore the ultrastructural characteristics of 5-HT innervation in several areas of different experimental models, mostly in rats, cats, and monkeys. The information collected provided insights on the intrinsic and relational morphological properties of 5-HT projections, revealing some notable features, partly shared with other monoaminergic systems.^51^ Serotonergic axons branch profusely and are characterized by numerous unmyelinated varicosities containing clear or dense core vesicles: those sites often lack the typical synaptic specializations or postsynaptic targets and only to a low extent seem to form proper synapses with specific neuronal targets. The presence of both junctional and nonjunctional varicosities can be considered solid evidence in support of the coexistence of WT and VT modalities of serotonin release.^44^ Moreover, in many regions of the CNS the frequency at which synaptic contacts are formed has been quantitatively evaluated, revealing a significant variability that suggests a differential role for WT and VT in different areas.

In some regions the asynaptic character of 5-HT innervation prevails, as it has been clearly observed for neocortex, striatum, and hippocampus. Serotonergic projections in the cerebral cortex arise from the dorsal and median raphe nuclei,^48,52−54^ and the fine structural characteristic of serotonergic axons has been surveyed in various regions of the cortex, by autoradiography and immunolabeling techniques and in different experimental models, such as rat, cat, and monkey.^55−57^ In these areas, with the exception of reports from one laboratory,^58^ it has been observed a lack of synaptic specializations in most of the 5-HT terminals. In particular, an assessment of the extent to which terminal-like varicosities display synaptic specializations has been performed in different regions of the adult rat cerebral cortex using 5-HT immunohistochemistry.^59^ The proportion of varicosities engaged in synaptic contacts (synaptic incidence) has been stereologically extrapolated to whole varicosities from thin sections, showing low values for both superficial (36%) and deep (27%) layers of the frontal cortex and for the occipital cortex (37%), while a slightly higher value (46%) has been estimated for the parietal cortex. In the striatum, as revealed by electron microscopy studies using autoradiographic labeling in rats and cats, serotonergic fibers that originate mostly from the dorsal raphe nucleus^52^ are to a large extent devoid of synaptic specializations.^60,61^ This notion has been corroborated in rats by the use of both autoradiography and 5-HT immunolabeling, which allowed estimation of the synaptic incidence to be as low as 10%.^62^ A comparable result (≈18%) was extrapolated from the scoring of SERT-immunolabeled varicosities obtained in the dorsolateral area of macaque putamen.^63^ Also the hippocampal formation, which receives a robust serotonergic innervation from both median and dorsal raphe nuclei,^54,64^ displays the prevalence of asynaptic terminals, the synaptic incidence being estimated to be around 12%,^55^ which includes the recently described synapse between serotonergic axons and primary cilia of CA1 pyramidal neurons.^65^

Serotonergic axons establishing synaptic contacts with specific targets are prominent in other brain regions such as the substantia nigra (SN), which receives 5-HT innervation from mesencephalic raphe nuclei.^52,66−69^ The SN represents one of the areas with the highest density of 5-HT innervation in the whole CNS, with the pars reticulata displaying significantly higher density as compared to pars compacta in both rat and monkey.^70,71^ Early ultrastructural studies demonstrated in this district the presence of synaptic contacts established by 5-HT immunoreactive fibers, mostly with dendrites.^66,72^ Subsequently, it has been shown a clear-cut difference in the characteristics of 5-HT innervation in the two subdivisions of SN in rat.^70^ In fact, in pars compacta, approximately 50% of the identified serotonergic terminal-like varicosities have been estimated to form synaptic contacts, whereas in pars reticulata, the total number of them shows synaptic membrane specializations. Those morphological characteristics suggest a predominant role for wiring transmission in SNr and a coexistence of both volume and wiring transmission in SNc.

The dorsal raphe nucleus (DRN) represents another notable region in which the morphological features of serotonergic neurons and their projections have been explored. This nucleus comprises a highly heterogeneous neuronal population, and it contains the cellular bodies of serotonergic neurons (B6–B7 groups) responsible for a large part of the 5-HT innervation to the forebrain.^52,54,73^ Though with a low density, the DRN of the rat is reached by 5-HT terminals that usually lack synaptic specializations^74^ and which could originate from other 5-HT groups, such as the caudal raphe nuclei, known to innervate the DRN.^75^ Moreover, early ultrastructural immunohistochemical analyses highlighted the presence of 5-HT immunoreactive small clear and dense-core vesicles in dendrites of DRN serotonergic neurons.^76−78^ In cats, some of those dendrites were observed to be involved in the formation of dendrodendritic synapses, while others lacked synaptic specializations, appearing as suitable sites for a possible extrasynaptic release.^77^ The existence of mechanisms of dendritic and also somatic 5-HT release is further sustained by direct ex vivo evidence. In particular, vesicular serotonin release from dendrites of DRN neurons has been demonstrated through a combination of three-photon microscopy and electron microscopy in rat living brain slices.^79^ In addition, perinuclear clusters of serotonergic vesicles have been identified in rat dorsal raphe sections and it has been shown a mechanism of nonsynaptic somatic release by potassium ions induced depolarization.^80^ This somatodendritic mechanism of serotonin release in the extracellular space, as well as the release from serotonergic terminals in the DRN, could be hypothesized to play a role in the mechanism of autoinhibition of 5-HT neurons’ firing, likely mediated by somatodendritic 5-HT_1A_ autoreceptors.^81^

On the whole, although the available ultrastructural data concerning serotonergic innervation have been collected over several decades by different research groups, using different approaches and experimental models, the results obtained converge in supporting the dualism of the 5-HT system and a role for both wiring and volume modalities in mediating serotonergic neurotransmission.

## 5-HT Receptors in Nonsynaptic Transmission

As previously mentioned, serotonin acts on its targets through multiple receptors.

In this regard, a first consideration relevant to the dualism of the serotonergic system concerns the types of receptors on which serotonin exerts its action. In fact, among the seven families of 5-HT receptors, only 5-HT_3_ receptors are ligand-gated cation channels,^82^ which mediate a fast postsynaptic excitatory response, with the other six all comprehending G-protein-coupled receptors.^83^ While ionotropic receptors are certainly suitable for rapid and specific synaptic communication, it is interesting to note that the general properties of metabotropic receptors, such as higher affinity for the ligand, slower response, and broader effects, fit better for the modulatory role of the neurotransmitter and are consistent with the properties of VT.

Among the notions that led to the definition of VT concept, a strong contribution has also been given by the observation of relative mismatches between the localization of neurotransmitters and their respective receptors in determined brain areas.^84,85^ This is the case for 5-HT_2A_ receptors, as it has been observed in a double-labeling immunohistochemistry study,^86^ in which the relationship between 5-HT fibers and 5-HT_2A_ immunoreactive targets has been evaluated at light microscopic level in the rat forebrain. Discrepancies have been found in the reciprocal localizations and density of those elements in the basal forebrain, as well as in cortical regions and in the hippocampus, suggesting that serotonin, possibly released by nonjunctional varicosities, reaches the 5-HT_2A_ receptors expressed by the targets through diffusion in the extracellular space. This hypothesis gains further support from the demonstration of the extrasynaptic localization of 5-HT_2A_ receptors on dendritic shafts of pyramidal and local circuit neurons in the rat prefrontal cortex (PFC).^87^ Indeed, the extrasynaptic localization, reported also for other 5-HT receptors, such as high affinity 5-HT_1_ receptors, is an aspect in line with the existence of serotonergic volume transmission in the CNS. For instance, the localization of 5-HT_1A_ receptors in sites devoid of synaptic specializations has been demonstrated on somata and dendrites of neurons in the dorsal raphe nucleus as well as in the hippocampal formation,^88,89^ two regions where nonsynaptic serotonergic varicosities presence is predominant. 5-HT_1B_ receptors have also been localized extrasynaptically, in association with unmyelinated preterminal axons in substantia nigra and globus pallidus, and it has been proposed a possible role for them in the modulation of axonal impulse conduction.^89,90^

Concerning the activity of extrasynaptic 5-HT receptors, a crucial issue to address is also whether they are exposed to a sufficiently high concentration of the neurotransmitter. Measurements of serotonin extracellular concentration in brain tissue are in line with the functionality of extrasynaptically located 5-HT receptors.^91^ In order to monitor dynamic changes in neurotransmitter concentration, compatible with events of release and reuptake, it is possible to exploit the fast-scan cyclic voltammetry (FSCV), a chemical-selective electroanalytical technique with high temporal resolution.^92^ Given that the dimensions of carbon fiber microelectrodes are consistently larger than the synaptic cleft, it is assumed that FSCV detects extrasynaptic concentrations of the neurotransmitter as it diffuses in the extracellular space following release. By the use of this technique in rat brain slices, following stimulation, the presence of extracellular serotonin has been detected in the dorsal raphe nucleus and in pars reticulata of the substantia nigra.^93,94^ According to the voltammetric measurements, serotonin could diffuse around 20 μm in the extracellular space, meaning that it can interact with many different extrasynaptic elements. Moreover, its concentration, evaluated after single stimulation pulses, closely matches the affinity of 5-HT_1_ receptors, which not only are highly expressed in both regions but, as mentioned above, have also proven to have an extrasynaptic localization.^88−90^ From the same study^94^ emerged an apparently contradictory result. In fact, the presence of extracellular serotonin, possibly implicated in volume transmission, has been detected in the substantia nigra pars reticulata, where ultrastructural analyses have revealed that all of the terminal-like serotonergic varicosities establish synaptic contacts with postsynaptic partners. This evidence suggests that the presence of extrasynaptic serotonin is due to a mechanism of spillover from the synaptic cleft, therefore prompting the existence of VT in brain districts structurally arranged for WT.

In summary, it can be assumed that although in some brain districts the ultrastructural features hint at a prevalence of one neurotransmission modality over the other, the two modalities may actually both coexist, displaying a higher variability than previously hypothesized.

## Serotonin–Glia Interplay via Volume Transmission

The likely presence of VT in brain regions structurally arranged for WT has a major impact on the interplay between serotonin and glial cells. In fact, in the CNS serotonin receptors are not expressed exclusively by neurons. Different functional 5-HT receptors are known to be expressed by distinct populations of glial cells, such as astrocytes^95^ and microglia,^96,97^ that do not establish synaptic contacts with 5-HT axons. Regardless of the existence of direct contacts with serotonergic fibers, there is strong evidence that glial cells respond to serotonin signaling to modulate important physiological and behavioral functions.

Serotonergic modulation has significant implications on the activity of astrocytes within the CNS. For instance, serotonin 1A receptors present on them promote the release of the neurotrophic factor S100β, involved in several cellular processes, such as cell cycle regulation and differentiation.^98,99^ In a mouse model for Parkinson’s disease, the activation of serotonin 1A receptors present on astrocytes has been reported to promote their proliferation and to upregulate antioxidative molecules, with a neuroprotectant effect.^100^ Another interesting effect of serotonin modulation has been found in a subset of hippocampal astrocytes expressing 5-HT_4_ receptors, whose activation is related to modifications of synaptic glutamate release.^101^ Serotonergic neurotransmission, through unspecified 5-HT receptors, has also been shown to positively modulate the astrocyte–neuron lactate shuttle, an important mechanism for the regulation of cerebral energy metabolism.^102^ In addition, 5-HT modulation of astrocytes has been correlated also to other effects, for example, in sleep^103^ and depression.^104−106^ Furthermore, besides being responsive to serotonergic signaling thanks to the expression of 5-HT receptors, astrocytes are also capable of uptaking extracellular serotonin^107,108^ thanks to the expression of the transporter SERT^109,110^ and other monoamine transporters, such as the organic cation transporter 3 (OCT3).^111^ Recently, a newly identified epigenetic role for serotonin uptake has been described.^112^ In particular, neuronal activity, through the increase of OCT3 expression and histone serotonylation in olfactory bulb astrocytes, has been demonstrated to enhance the levels of astrocytic GABA biosynthesis and release, with effects on olfactory processing and behavior.

Concerning microglia, which expresses 5-HT_2B_ receptors, there is compelling evidence supporting the importance of serotonergic modulation through its activation during rodent brain development.^113^ In this regard, selective inactivation of 5-HT_2B_ receptor in microglia early in life has been correlated to prolonged neuroinflammation and increased sick behavior in adult, supposedly due to defects in microglia maturation or alteration in its developmental interactions with nearby neurons.^114^ Its ablation during a critical postnatal developmental period has also been shown to determine the alteration of neuronal circuits and behavioral effects in the adult, impairing sociability and flexibility.^115^ The influence of serotonin on microglia activation directly links 5-HT to the regulation of neuroinflammation and events occurring in neurodegenerative diseases such as ALS and Parkinson’s disease or following neuronal damage.^114,116−119^

As previously mentioned, glial cells do not receive synaptic contacts from serotonergic fibers. Consequently, in light of the modalities of neurochemical interaction between glial cells and neurons, it is reasonable to expect that those glial-expressed 5-HT receptors are activated through VT by extrasynaptic serotonin originating from nonjunctional varicosities or synaptic cleft spillover.

On the whole, the presence of 5-HTRs on glial cells represents strong evidence for the existence of serotonergic volume transmission in the CNS. Moreover, although future experiments are mandatory to associate specific roles to either WT or VT, the activity of serotonin on glial cells clearly demonstrates not only the existence but also the relevance of a dual modality of serotonergic neurotransmission.

## Role of Serotonin Transporter in Nonsynaptic Transmission

In the perspective of the wide-ranging effects of serotonin due to the presence of volume transmission, a final critical aspect to consider concerns the removal of the neurotransmitter from the extracellular space. The energy-dependent mechanism of neurotransmitter reuptake mediated by the high-affinity serotonin transporter SERT is crucial for the regulation of serotonergic neurotransmission.^120^ In addition to SERT, there are also other less specific transporters with lower affinity, such as organic cation transporters (OCTs), which are broadly expressed in the brain and can participate in the clearance of serotonin.^121^

As for other monoamine transporters, such as norepinephrine and dopamine transporters,^122−124^ SERT has been directly localized via immunocytochemical EM studies on the axolemma at extrasynaptic sites in different districts of the CNS.^110,125−127^ Specifically, functional 5-HT transporter has been identified in perisynaptic areas and on intervaricosity axonal segments devoid of synaptic specializations in rat dorsal raphe, corpus callosum, medial forebrain bundle, cingulum bundle, and cingulate cortex.^127^ This extrasynaptic localization of SERT is indicative of the fact that this important part of the regulation of serotonergic signaling takes place outside of synaptic contacts, in accordance with the voltammetrical measurements.^94^ Those data are consistent with the role of SERT in the clearance of the extracellular space from serotonin molecules involved in volume transmission.

The relevance of SERT-mediated uptake of extrasynaptic serotonin already emerges during early brain development, as perinatal exposure to selective serotonin reuptake inhibitors (SSRIs) is related to the emergence of paradoxical depressive-like and anxiety-like behaviors in the adult life.^128−131^ While in the adult brain SERT is mostly expressed by 5-HT producing neurons, during development it displays an early and dynamic expression in nonserotonergic neurons,^132,133^ as revealed by the use of SERT-Cre mouse model.^134^ This spatiotemporal pattern of SERT expression is likely required for a fine regulation of serotonin extracellular content. In order to address this hypothesis, the functional significance of transient SERT expression during postnatal development has been specifically investigated.^135^ Results confirmed that SERT is required to maintain the serotonin homeostasis for the proper formation of descending prefrontal circuits targeting DRN, which are involved in stress response. Specifically, it has been shown that transient presence SERT in PFC pyramidal neurons is required to prevent excessive activation of 5-HT_7_ receptors in the same district.^136^

Overall, the activity of transiently expressed SERT transporter in nonserotonergic neurons during critical developmental periods appears as a plausible modality through which extrasynaptic morphogenetic gradients of serotonin can be shaped, in order to control to what extent volume transmission regulates the formation of connections in the wired brain.

## Conclusions, Open Questions, and Future Perspectives

Since the discovery of central 5-HT neurons, compelling evidence regarding the dualism of serotonergic transmission has been collected at several different levels, building a complex framework in which serotonin appears to exert its activity through both wiring and volume transmission.

**Figure 2:**
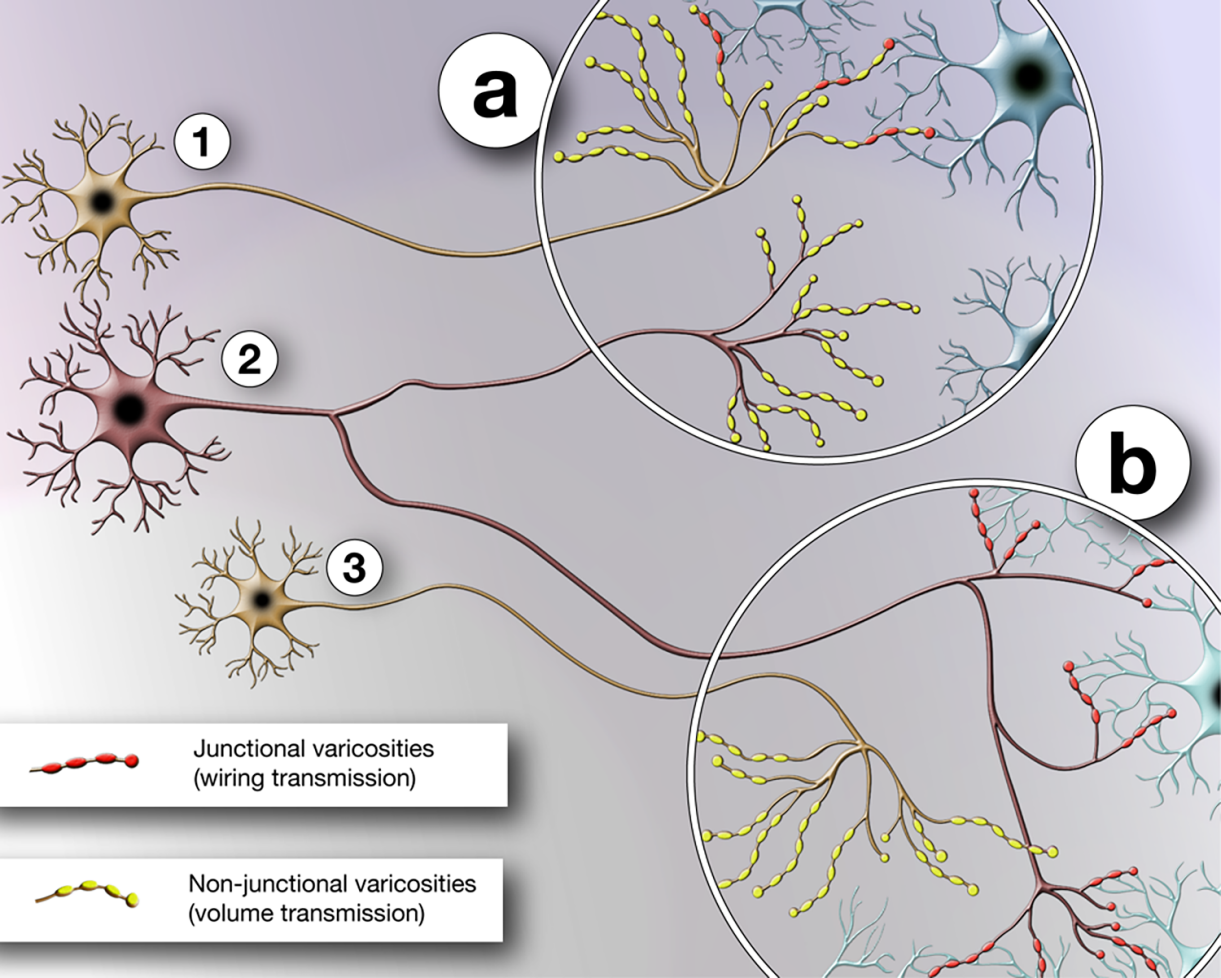
Schematic representation of the hypothetical distribution of the two modalities of neurotransmission. In the scheme three serotonergic neurons are represented with neuron 1 projecting to target area (a) and displaying both wiring transmission and volume transmission, neuron 2 sending projections to target areas (a) and (b) where it displays volume transmission and wiring transmission modality, respectively, and neuron 3 projecting to target area (b) showing exclusively volume transmission.

While in the serotonergic system both a rapid and precise way of communication and a broader and slower modality coexist, their specific role in the regulation of serotonin-mediated developmental, physiological, and behavioral effects has yet to be clarified and several questions are still open. As discussed above, in several brain areas, the anatomical data suggest the prevalence of either wiring or volume transmission, based on the relational characteristics of the serotonergic innervation. However, due to the extraordinarily high complexity of the serotonergic wiring, it remains to elucidate whether along a single axonal filament a promiscuous serotonin release modality is present. In such a scenario, as distinct brain areas may receive serotonergic axons from the same neuron,^137,138^ it is even harder to establish whether the single serotonergic neuron uses the same modality of neurotransmission in different areas (Figure 2). It therefore emerges as a matter of future research whether serotonergic neurons are predetermined for volume transmission, wiring transmission, or both modalities. If, as it seems, volume transmission plays a relevant role in serotonergic signaling, this dualism could have important consequences on aspects implicated not only with the physiological activity of this system but also with its pathological dysfunctions as well as with therapeutic interventions. For instance, in light of a sizable extrasynaptic localization of SERT, it is tempting to speculate that the inhibition of serotonin reuptake via SSRIs administration or the use of abuse drugs such as MDMA/Ecstasy could elevate the extrasynaptic serotonin content, therefore ascribing, at least in part, to volume transmission signaling the therapeutic effect or damaging effects, respectively.^139^ Therefore, as this dualism can have a strong impact on serotonergic neurotransmission, in the perspective of further understanding the role of serotonin in the CNS, it is mandatory to widen our knowledge regarding this aspect by applying new technologies and molecular approaches. In this sense, a possible approach could be to exploit the characteristics of retrograde tracers based on viral vectors, such as the rabies virus, which have the capacity to selectively transduce neurons at the presynaptic terminal.^140^ The use of such a tool to trace the serotonergic neurons would specifically allow drawing a map of the serotonergic neurons displaying wiring transmission. The combination of retrograde tracers with the capacity to label fibers irrespective of the presence of synaptic specializations, such as retrobeads,^141^ rAAV,^142^ or pseudotyped rabies virus,^143^ would allow us to take a step forward in understanding, for instance, whether serotonergic neurons projecting to a specific brain region use in that district both modalities or only/predominantly the volume transmission.
